# Blockade of Inhibitors of Apoptosis Proteins in Combination with Conventional Chemotherapy Leads to Synergistic Antitumor Activity in Medulloblastoma and Cancer Stem-Like Cells

**DOI:** 10.1371/journal.pone.0161299

**Published:** 2016-08-18

**Authors:** Shu-Mei Chen, Ying-Ying Li, Chiao-Hui Tu, Nicole Salazar, Yuan-Yun Tseng, Shiang-Fu Huang, Ling-Ling Hsieh, Tai-Ngar Lui

**Affiliations:** 1 Graduate Institute of Clinical Medical Sciences, College of Medicine, Chang Gung University, Tao-Yuan, Taiwan; 2 Department of Neurosurgery, Wan Fang Hospital, Taipei Medical University, Taipei, Taiwan; 3 Sheila and David Fuente Graduate Program in Cancer Biology, University of Miami Miller School of Medicine, Miami, Florida, United States of America; 4 Department of Neurosurgery, Shuang Ho Hospital, Taipei Medical University, Taipei, Taiwan; 5 Department of Surgery, School of Medicine, College of Medicine, Taipei Medical University, Taipei, Taiwan; 6 Department of Public Health, College of Medicine, Chang Gung University, Tao-Yuan, Taiwan; 7 Department of Otolaryngology-Head and Neck Surgery, Chang Gung Memorial Hospital, Chang Gung University, Taoyuan, Taiwan; Columbia University, UNITED STATES

## Abstract

**Background:**

Medulloblastoma (MB) is the most common pediatric primary malignant brain tumor. Approximately one-third of MB patients succumb to treatment failure and some survivors suffer detrimental side effects. Hence, the purpose of this study is to explore new therapeutic regimens to overcome chemotherapeutic agent resistance or reduce chemotherapy-induced toxicity.

**Methods:**

We detected the expression of inhibitors of apoptosis proteins (IAPs) in MB and CD133+ MB cell lines and MB tissues using immunoblotting and immunohistochemical staining. The antitumor effects of inhibitors against IAPs on MB or CD133+ MB cells were evaluated by MTT assay, Annexin V/PI analysis, and caspase-3/7 activity. Autophagy was assessed by the conversion of light chain (LC) 3-I to LC3-II and Cyto-ID autophagy detection kit.

**Results:**

MB cells showed higher expression of IAPs compared to normal astrocytes and normal brain tissues. Conventional chemotherapeutic agents combined with small-molecule IAP inhibitors (LCL161 or LBW242) showed a synergistic effect in MB cells. Combined treatments triggered apoptosis in MB cells through activation of caspase-3/7 and autophagic flux simultaneously. In addition, we found that CD133+ MB cells with features of cancer stem cells displayed higher levels of X-linked inhibitor of apoptosis (XIAP) and cellular inhibitor of apoptosis 1/2 (cIAP1/2), and were hypersensitive to treatment with IAP inhibitors.

**Conclusions:**

These results shed light on the biological effects of combination therapy on MB cells and illustrate that IAP inhibitors are more effective for CD133+ stem-like MB cells.

## Introduction

Medulloblastoma (MB), an embryonic tumor of the cerebellum, is the most common malignant childhood brain tumor, comprising 15–30% of intracranial tumors in the pediatric population [1] with a peak incidence of 3–9 years of age [2]. It is a highly invasive and fast growing tumor, and frequently metastasizes to different locations within the brain or spinal cord. Although multiple therapeutic modalities have been developed, 15–40% of MB patients have a high risk of dying from tumor recurrence [3–7]. Therefore, developing new effective therapeutic regimens, which can prolong survival and reduce the impact of chemodrug-induced toxicity, is critical for MB patients. Over the past two decades, the conventional chemotherapeutic agents for treating MB patients include vincristine and cisplatin [7–10]. Unfortunately, these drugs have harmful side effects and give rise to resistance. Numerous strategies have been provided to overcome drug resistance by targeting survival mechanisms, such as autophagy-induced stable diseases, anti-apoptotic proteins, efflux pump-reduced intratumor chemodrugs, and cancer stem cells (CSCs).

One of the mechanisms leading to chemotherapy resistance is up-regulation of X-linked inhibitor of apoptosis protein (XIAP) and cellular inhibitor of apoptosis 1/2 (cIAP1/2). In melanoma and MB cells, downregulation of XIAP and cIAP1/2 is associated with sensitivity to chemotherapies [11]. Recent studies have shown that inhibitors against inhibitors of apoptosis proteins (IAPs) are able to overcome drug resistance, and combination with different chemotherapies can induce type I cell death via activation of caspase-3, 7, and 9 *in vitro* and *in vivo* [12]. Another cell death, autophagic cell death (type II cell death), has been discovered in Bax/Bak deficient mouse embryonic fibroblasts (MEFs) following treatment with apoptotic stimuli [13]. The presence of anti-autophagy inhibitors or silencing autophagic molecules including Atg5 and Atg6 can rescue MEFs from undergoing autophagic cell death and improve clonogenicity. Nevertheless, several studies indicated that during deprivation of nutrients, depletion of growth factors, or targeted treatments, autophagy leads cells towards cell survival via degradation of macromolecules [14,15]. They suggested that autophagy may be a protective mechanism to refrain cells from undergoing mitochondrial polarization and mitochondria-dependent cell death [14,15]. Hence, whether autophagy enhances cell death or cell survival remains unclear and controversial.

Zanini *et al* suggested that subsets of MB cells with stemness markers such as CD133, CD44, Oct4, and Nanog are considered cancer stem cells or cancer stem-like cells [16]. Recent data indicate that cancer stem-like cells exhibit resistance to chemotherapies and radiation, which leads to treatment failure in neuroblastoma [5] and MB [17]. In neuroblastoma, CD133+ cells are chemo-resistant and can be enriched *in vivo* following treatment with doxorubicin, etoposide, or cisplatin [18,19]. In MB, cancer stem-like cells are resistant to TNF-related apoptosis-inducing ligand (TRAIL)-induced radiosensitivity and TRAIL-induced apoptosis due to high expression of anti-apoptotic genes including Bcl-2 and c-FLIP [17]. Another study also demonstrated that the combination of XIAP inhibition and TRAIL is able to bypass overactive Bcl2-mediated resistance to TRAIL, and in turn suppress the growth of pancreatic cancer *in vitro* and *in vivo* [20]. Therefore, we hypothesized that IAP inhibitors may be capable of improving sensitivity to chemotherapy particularly in CD133+ stem-like MB cells. Although there are no references showing that CD133+ stem-like cells are more resistant to chemotherapies in MB, they were reported to result in radiation resistance [21]. Thus, combination of chemotherapies and IAP inhibitors may be a legitimate alternative option for MB patients.

The IAP inhibitors LCL161 and LBW242 are structural mimics of Smac/DIABLO, targeting XIAP and cIAP1/2. LBW242 has a synergistic effect on glioma when combined with temozolomide [12], and has the capacity to pass the blood-brain barrier in animal models [22]. In this study, our results demonstrated that these IAP inhibitors attenuate broader and novel mechanisms that contribute to chemoresistance in MB cells. We also discovered that combined treatments (cisplatin or vincristine with IAP inhibitors) have cytotoxicity and trigger concomitant type I and type II cell death through activation of caspase-3/7 and autophagic flux in MB cells. The effect of IAP inhibitors diminishing cell proliferation and inducing cell death not only occurs in MB cells but also in CD133+ stem-like MB cells, which express higher levels of XIAP and cIAP1/2. These results suggest that IAP inhibitors are potential candidates to treat MB cells and CD133+ stem like MB cells.

## Materials and Methods

### Tissue array and Immunohistochemistry

The tissue array for MB (BC17012b) was bought from US Biomax, Inc. and stated that each specimen collected from any clinic was consented by both hospital and individual. Discrete legal consent was obtained and the rights for research purposes or further commercialized use were waived (http://www.biomax.us/support.php). Tissue sections were deparaffinized in xylene and absolute ethanol, retrieved with citric acid, and then incubated with 3% hydrogen peroxide to eliminate endogenous peroxidase activity. Immunohistochemical staining was performed using specific rabbit polyclonal antibodies against XIAP (10037-1-lg, Proteintech, 1:200), and the UltraVision^™^ Quanto Detection System (Thermo Fisher Scientific). Slides were then counterstained with hematoxylin, and mounted with Permount (Fisher Chemical).

### Cell lines, chemicals, and cell culture

Human MB cell lines, DAOY and D283MED, were purchased from the American Type Culture Collection and cultured in minimum essential medium (MEM, Life Technologies) supplemented with 10% fetal bovine serum (FBS, Life technologies) and 1% penicillin streptomycin (Life Technologies), and incubated at 37°C in 5% CO_2_. The normal Human Astrocyte-hippocampal cell line (HA-h, Sciencell Research Laboratories, catalog #1830) was kindly provided by Dr. Ruei-Ming Chen (Taipei Medical University, Taiwan), and cultured in Astrocyte Medium (Sciencell Research Laboratories).

The IAP inhibitors LCL161 and LBW242 were kindly provided by Novartis Pharma. Stock solutions of LCL161 and LBW242 were prepared in dimethyl sulfoxide (DMSO, Sigma-Aldrich), stored at −20°C, and diluted in fresh medium immediately prior to use. Vincristine (Teva Pharmaceuticals) and cisplatin (Teva Pharmaceuticals) were obtained from Wanfang Hospital pharmacy (Taipei) and diluted in saline prior to use. The caspase-3 inhibitor (Z-DEVD-FMK, Selleckchem) was dissolved in DMSO (10 mM stock) and diluted in sterile saline immediately before use. Chloroquine (N^4^-(7-Chloro-4-quinolinyl)-N^1^,N^1^-dimethyl-1,4-pentanediamine diphosphate salt, Sigma-Aldrich) was dissolved in sterile nuclease-free water for storage and usage.

### Isolation and culture of CD133+ MB cells

DAOY and D283MED cells (2 × 10^5^ cells/ml) were stained with phycoerythrin (PE)-conjugated anti-human CD133 antibody (Miltenyi Biotec). Thereafter, CD133+ cells were sorted, collected by flow cytometry sorting (Becton Dickinson FACSAria III), and cultured in DMEM-F12 (Life Technologies) with 10ng/ml bFGF (ProSpec), 10ng/ml EGF (ProSpec), and 1% N2 (Life Technologies).

### Cell viability assay

Cells were seeded into 96-well plates at 0.5–1 x 10^4^/well and incubated overnight. After treatment with these agents for 72 hr, cells were incubated with 50 μl 3-(4, 5-dimethylthiazol-2-yl)-2, 5 -diphenyltetrazolium bromide (0.5 mg/ml MTT, Sigma) for 4 hr, and MTT-metabolized formazan was dissolved in 180 μl isopropanol-HCl. Finally, the optical density (OD) of the solution was determined with a spectrometer at a wavelength of 570 nm. The cell viability was calculated by a formula: (OD of experimental well/OD of control) × 100%. Three replicates were included for each experiment.

### Preparation of cell lysates and immunoblotting

Cells pellets were suspended in RIPA buffer (150 mM NaCl, 1 mM EDTA, 1% Nonidet P-40, 0.5% Sodium deoxycolate, 0.1% SDS, and 50 mM Tris–HCl) supplemented with protease inhibitors and phosphatase inhibitors. Cell lysates were prepared after homogenization and centrifuged at 14000 rpm at 4°C for 15min. Total proteins were separated using 8%-12% sodium dodecyl sulfate polyacrylamide gel electrophoresis (SDS-PAGE), and then transferred to nitrocellulose membranes (Millipore). Afterwards, membranes were blocked with 5% non-fat milk prepared in TBS-T containing 10 mM Tris-HCl, pH 7.4, 150 nM NaCl, and 0.01% (v/v) Tween 20 for 1 hour, washed with TBS-T, then probed with antibodies against XIAP, cleaved PARP, PARP, and LC3-I/II (Cell Signaling Technology); cIAP1/2, GAPDH (Santa Cruz Biotechnology) at 4°C overnight. After TBS-T washing, the membranes were incubated with goat anti-rabbit- and anti-mouse- IgG coupled with horse-radish peroxidase (Santa Cruz Biotechnology) at room temperature for 2 hrs. Finally, detection was completed using enhanced chemiluminescence substrate (Millipore).

### Annexin V/PI analysis

Cells were harvested following drug treatments and then stained using an Annexin V/PI double staining kit (Calbiochem) according to the manufacturer’s instructions. Apoptotic cells were distinguished from control non-apoptotic cells using a Beckman Coulter Epics XL flow cytometer.

### Measurement for Caspase-3/7 Activity

Cells were harvested and caspase-3/7 activity was examined using a CaspaTag Caspase-3/7 in situ assay kit (Millipore) according to the manufacturer’s instructions. Briefly, 1 x 10^6^ cells were resuspended in 300 μl of medium with different treatments and combined with 10 μl of freshly prepared 30x FAM-DEVD-FMK (FLICA) reagent at 37°C for 1 hr. Caspase-3/7 activities were analyzed by FACS.

### Detection of autophagy

Cells were harvested and autophagy flux was determined using a Cyto-ID autophagy detection kit (Enzo Life Sciences) according to the manufacturer’s instructions. Cells were then analyzed by FACS. Data were presented as the mean fluorescence intensity of Cyto-ID divided by the mean forward scatter of the cells.

### siRNA transfections

siRNAs targeting cIAP1/2, XIAP, and non-targeting siRNAs were purchased from TOOLS, Taiwan. MB cells were cultured in antibiotic-free medium for 24 hr prior to transfection with 10 nM siRNA targeting cIAP1/2, XIAP, or corresponding non-targeting controls. Transfections were carried out in antibiotic-free medium using INTERFERin transfection reagent (Polyplus).

### Statistical Analysis

Statistical analysis was carried out using Microsoft Excel software and SigmaPlot 10.0 to assess the differences between experimental groups. Statistical significance was analyzed by Student’s t-test and shown as a p-value. The p-value < 0.05 referred to statistical significance.

## Results

### High expression of IAPs appears in human MB tissues and cell lines

**Fig 1:**
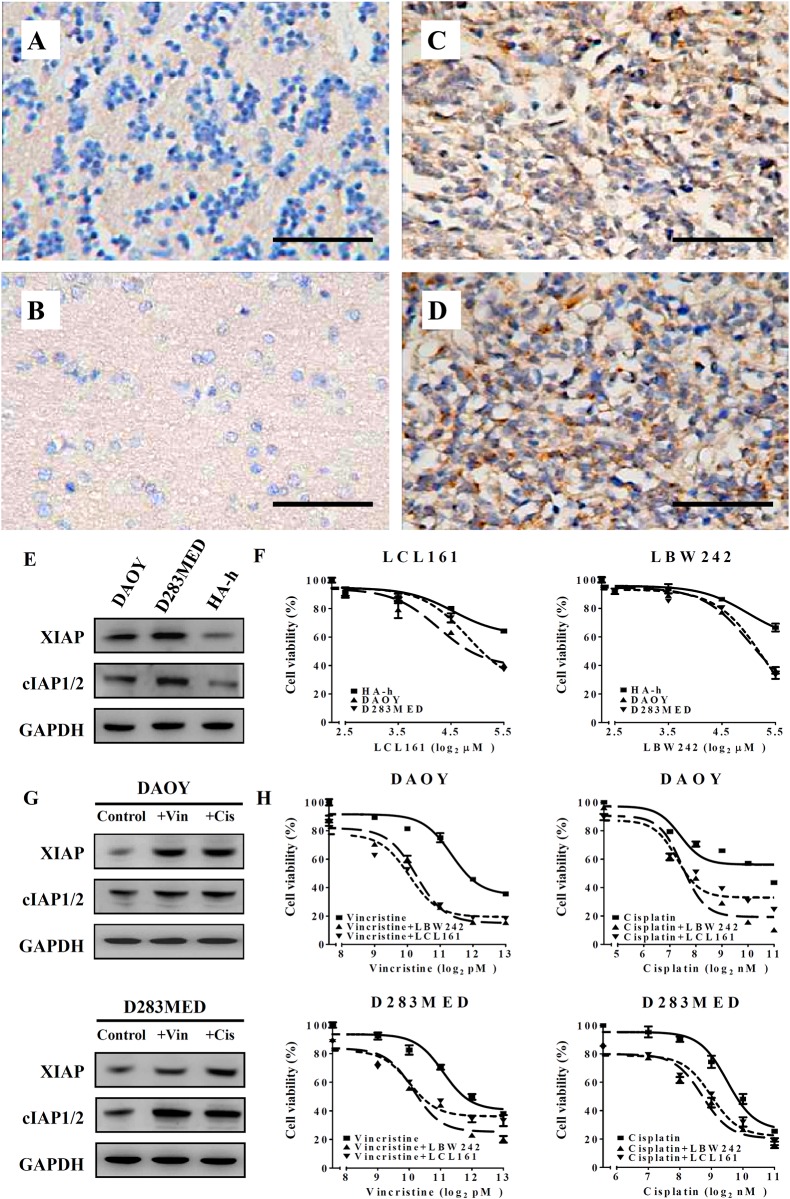
XIAP is specifically expressed in medulloblastoma (MB) tissue and both XIAP and cIAP1/2 can be enhanced by addition of chemotherapeutic agents to render MB cells hypersensitive to IAP inhibitors. XIAP levels were detected by immunohistochemical staining in normal cerebellum tissue (A), normal cerebrum tissue (B), and MB tissues (C, D). Bar scale represents 100 μm. (E) The levels of XIAP and cIAP1/2 in MB cell lines (DAOY and D283MED) and normal astrocytes (HA-h) were determined by immunoblotting. (F) DAOY, D283MED, and HA-h cells were treated with different concentrations of LCL161, LBW242, and DMSO (control) for 72 hr. Cell viability was analyzed by MTT assay. (G) The cell lysates were collected from MB cells and subjected to immunoblotting after treatment with vincristine (1.25 nM for DAOY, 2.5 nM for D283MED) or cisplatin (0.3125 μM for DAOY and 0.625 μM for D283MED) for 72hr. (H) DAOY and D283MED cells were treated with the indicated concentrations of vincristine or cisplatin in combination with LBW242 (10 μM) or LCL161 (10 μM). The cell viability was determined by MTT after these treatments for 72hr. The data are presented as the mean ± SEM of three independent experiments.

To verify whether MB tumors and cell lines have higher levels of IAPs, we determined XIAP and cIAP1/2 levels using immunoblotting. The results showed higher expression of XIAP and cIAP1/2 in MB cells lines (DAOY and D283MED) relative to normal astrocytes (HA-h) (Fig 1E). Additionally, we also confirmed XIAP expression in triplicate cores of tumors from twenty MB patients and three normal brain tissues. Approximately 75% of MB tissues but none of the normal brain tissues had XIAP expression in the cytoplasm (Fig 1A–1D).

### Synergistic effect of conventional chemotherapeutic agents combined with IAP inhibitor on MB cells

Since we found that MB cells showed high expression of IAPs, we next examined whether MB cells are sensitive to IAP inhibitors (LCL161 and LBW242). The results showed that LCL161 or LBW242 could inhibit 50% cell viability at 30 μM in MB cells while and a mild inhibitory effect on HA-h cells (Fig 1F).

Both vincristine and cisplatin are adjuvant chemotherapeutic agents utilized for the treatment of diverse cancer types including MB. We confirmed that XIAP and cIAP1/2 were up-regulated in DAOY and D283MED cells following treatment with vincristine or cisplatin (Fig 1G). This result was in accordance with another previous study [23]. To investigate the effect of IAP inhibitors as an add-on therapy to vincristine or cisplatin treatment in MB cells, we examined the cell viability of DAOY and D283MED cells using MTT assay. We treated the cells with a fixed concentration of IAP inhibitors (10 μM LCL161 or LBW242) and combined it with different concentrations of vincristine or cisplatin. Combination with IAP inhibitors diminished cell viability over 20% as compared to single treatment with cisplatin or vincristine (Fig 1H). In addition, a 2 to 5-fold decrease in ED_50_ of vincristine or cisplatin appeared in DAOY and D283MED cells when combined with LCL161 as well as LBW242 compared to vincristine or cisplatin alone (Fig 1H and Table 1). To examine whether the combination had a synergistic effect, a combinatorial index (CI) analyzed by CompuSyn software was applied, and a CI < 1 indicated the synergistic effect of conventional chemotherapy drugs combined with IAP inhibitors on MB cells. As we expected, LBW242 or LCL161 synergized with the anti-tumor effects of cisplatin or vincristine in both DAOY and D283MED cells (Table 2).

**Table 1:** ED50 values of chemotherapeutic agents alone or in combination with IAP inhibitors in DAOY and D283MED cells.

Cell line	Treatment	ED50	p-value*
DAOY	Vincristine	5.5±0.61nM	
	Vincristine+LBW242	1.3±0.18nM	0.0113
	Vincristine+LCL161	1.1±0.20nM	0.0105
	Cisplatin	1.8±0.10uM	
	Cisplatin+LBW242	0.43±0.02uM	0.0028
	Cisplatin+LCL161	0.3±0.13uM	0.0059
D283MED	Vincristine	5.7±0.47nM	
	Vincristine+LBW242	1.8±0.32nM	0.0105
	Vincristine+LCL161	2.4±0.11nM	0.0105
	Cisplatin	1.2±0.25uM	
	Cisplatin+LBW242	0.5±0.03uM	0.059
	Cisplatin+LCL161	0.6±0.02uM	0.0774

*Comparing to treatment with vincristine or cisplatin

**Table 2:** Combination Index (CI) of conventional chemotherapeutic agents (at ED50) combined with IAP inhibitors in medulloblastoma cells.

Cell line	Combination Therapy	Combination Index at ED50
DAOY	Vincristine + LBW242	0.57
	Vincristine + LCL161	0.63
	Cisplatin + LBW242	0.35
	Cisplatin + LCL 161	0.54
D283MED	Vincristine + LBW242	0.55
	Vincristine + LCL161	0.66
	Cisplatin + LBW242	0.66
	Cisplatin + LCL 161	0.83

Note: combinatorial index (CI) was calculated using CompuSyn software. CI = 1 (±0.2) indicates an additive effect, CI > 1 indicates an antagonistic effect, and CI < 1 indicates the synergistic effect of combination therapy on medulloblastoma.

### Combination of IAP inhibitors and chemotherapeutic agents results in concomitant induction of autophagy and apoptosis in MB cells

**Fig 2:**
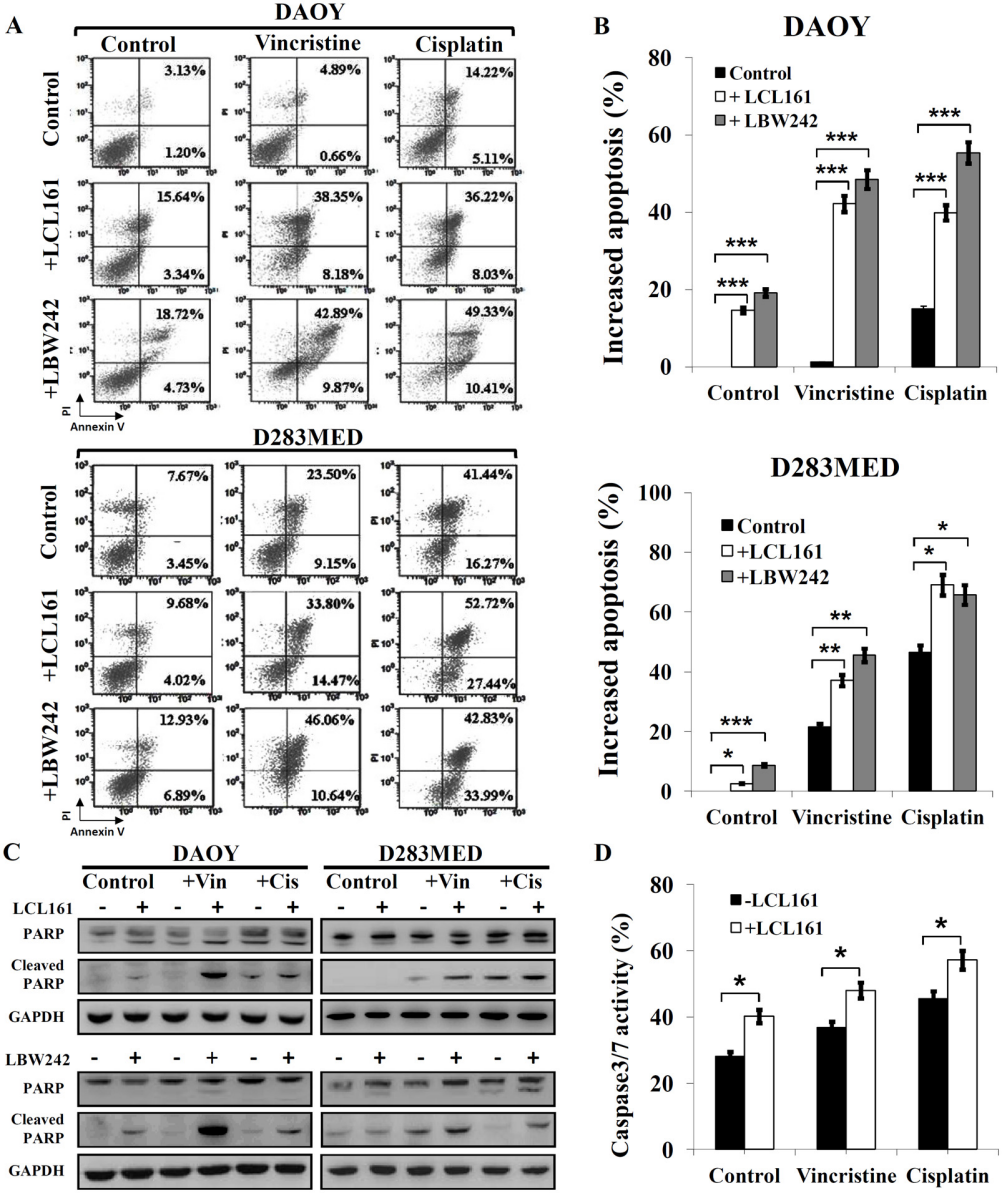
Addition of IAP inhibitors enhances chemotherapy-induced apoptosis in MB cells. DAOY and D283MED cells were treated with vincristine (1.25 nM for DAOY, 2.5 nM for D283MED) or cisplatin (0.3125 μM for DAOY, 0.625 μM for D283MED), or combination of the chemotherapeutic drug with an IAP inhibitor (LCL161 or LBW242, 10 μM) for 72 hr. (A)(B) Apoptosis was assessed by Annexin V/PI and FACS. The apoptotic proportions (Annexin V positive cells) are shown in bar graphs. (C) MB cells were treated with these chemotherapeutic agents combined with or without IAP inhibitors for 72 hr, and cell lysates were then subjected to immunoblotting analysis. (D) DAOY cells were treated with vincristine or cisplatin only, or in combination with LCL161 for 72 hr. The bar graph represents Caspase-3/7 activity, which was analyzed by FACS. The error bars represent mean ± SEM (*p < 0.05, **p < 0.001, and ***p < 0.005).

To clarify whether MB cells undergo apoptosis after IAP inhibitor treatment or combination therapy, we detected apoptotic cells using Annexin V/PI and FACS. The results revealed that LCL161 or LBW242 (10 μM) slightly increased apoptosis (15–19% in DAOY and 3–9% in D283MED), and vincristine (1.25–2.5 nM) and cisplatin (0.31–0.62 μM) induced approximately 2% and 15% of apoptotic proportions in DAOY cells, and approximately 22% and 47% in D283MED cells (Fig 2A and 2B), respectively. Treatment with LCL161 or LBW242 (10 μM) dramatically increased (20–40%) cisplatin- or vincristine- induced cell apoptosis in these two cell lines. The add-on benefit of LCL161 or LBW242 to these chemotherapeutic agents was significant in MB cells, while not in HA-h cells (S1 Fig).

Activation of apoptosis was also represented by activity of caspase-3/caspase-7 and protein levels of cleaved poly (ADP-ribose) polymerase (PARP), a downstream molecule of the caspase cascade. As shown in Fig 2C and 2D, IAP inhibitors (LCL161 or LBW242) enhanced the levels of cisplatin- or vincristine-induced cleaved PARP and increased chemotherapy drug-induced caspase-3/7 activity by 15%. Conversely, addition of the caspase-3 inhibitor (Z-DEVD-FMK, 40 μM) could rehabilitate the combination of vincristine and LCL161-induced apoptotic proportions by 7.3% and 18.4%, and the combination of cisplatin and LCL161-induced cell death by 23.0% and 31.9% in DAOY and D283MED cells, respectively (S2A and S2B Fig).

**Fig 3:**
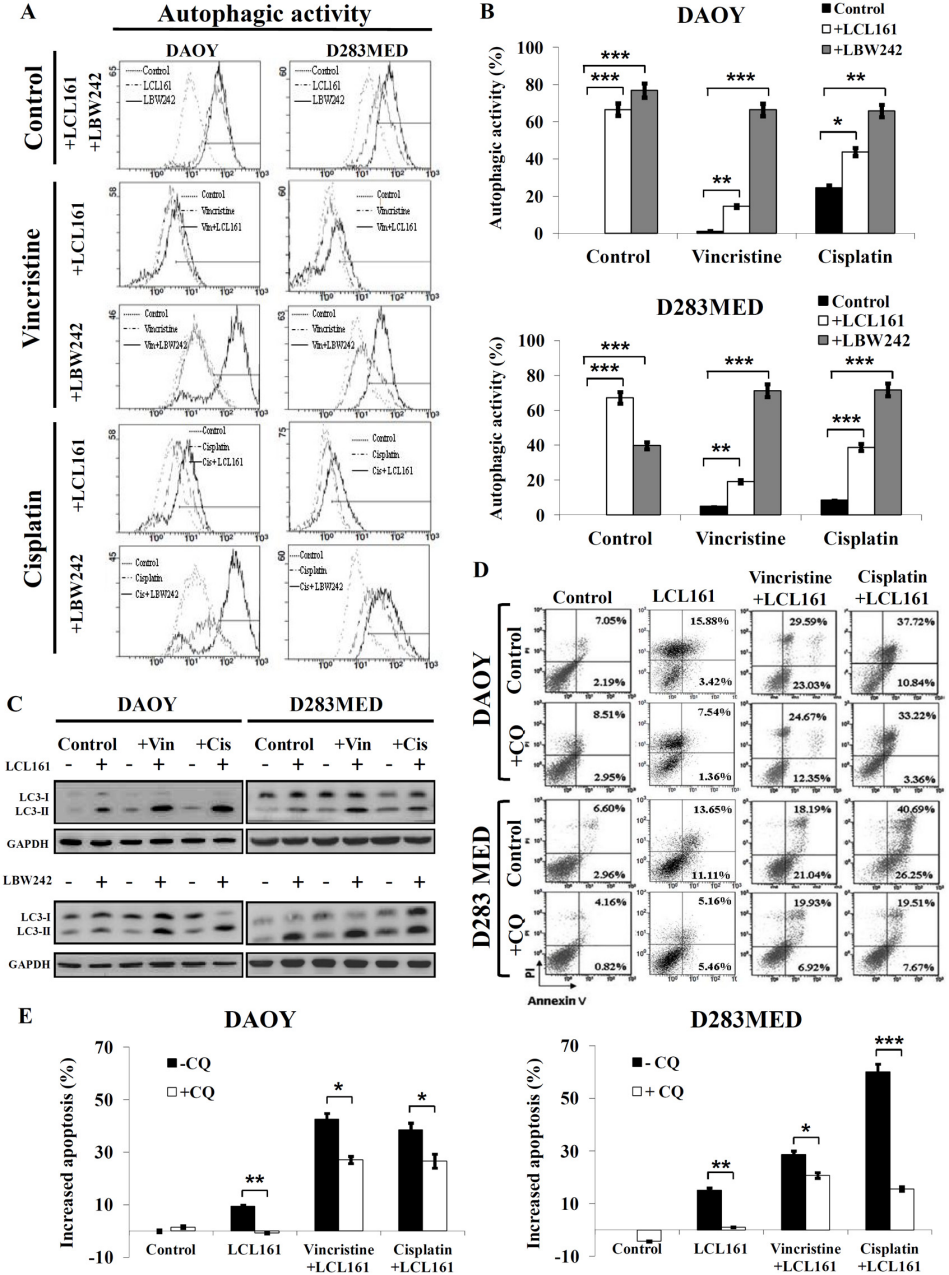
Combination of IAP inhibitors and chemotherapeutic agents promotes autophagy-dependent apoptosis. (A) DAOY and D283MED cells were treated with chemotherapeutic agent (vincristine or cisplatin) only or in combination with DMSO, LCL161 (10μM) or LBW242 (10μM) for 72 hr. Autophagic flux was determined by cyto-ID and analyzed by FACS. (B) Autophagic flux was based on percentage of right shift and has been shown in bar graphs (*p < 0.05, **p < 0.001, and ***p < 0.005). (C) LC3-I/II conversion was determined by immunoblotting after these treatments for 72 hr. (D) (E) The autophagy inhibitor (chloroquine, 10 μM) reduced combination therapy-induced apoptosis, as determined by Annexin V/PI and FACS. The bar graphs show the proportions of Annexin V positive cells (*p < 0.05, **p < 0.001, and ***p < 0.005).

As previously mentioned, it has been known that cells can undergo apoptosis through type I or type II cell death mechanisms. To investigate whether IAP inhibitors are capable of initiating autophagic flux, we determined autophagosome formation using cyto-ID and quantitated this by FACS. In DAOY and D283MED cells, LCL161 or LBW242 (10 μM) increased the right-shift intensity of cyto-ID by 40–78%, whereas cisplatin or vincristine barely induced < 25% shift. Combined treatments steadied the autophagic activity (Fig 3A and 3B). Furthermore, LC3 has been regarded as a hallmark of autophagy, and conversion of LC3-I to LC3-II through proteolytic cleavage and lipidation are representative of induction ofautophagosome formation. Hence, we observed conversion of LC3-I to LC3-II using immunoblotting. Subsequent to incubation with IAP inhibitor (10 μM) or combined treatments for 72hr, the levels of cleaved LC3 (LC3-II) were upregulated compared to treatment with cisplatin, vincristine, or DMSO alone (Fig 3C). As reported previously, whether autophagy results in cell death or cell survival is unknown. Therefore, we hypothesized that IAP inhibitors combined with cisplatin or vincristine induced type II cell death via autophagic flux. We treated MB cells with IAP inhibitors and chemotherapeutic drug (cisplatin or vincristine) with the anti-autophagy agent chloroquine (CQ) to examine whether inhibition of autophagy is able to rescue cells from combined treatment-induced cell death. We found that CQ (10 μM) not only reduced combined treatment of vincristine and LCL161-induced apoptosis by 15.6% and 7.85%, but also reversed combined treatment of cisplatin and LCL161-induced cell death by 12.0% and 44.37% in DAOY and D283MED cells, respectively (Fig 3D and 3E). Hence, we suggest that IAP inhibitors give rise to concomitance of type I (apoptosis) and type II (autophagy) cell death, and in turn dramatically diminish cell survival in MB cells.

### Treatment with IAP inhibitors or silencing XIAP or cIAP1/2 significantly enhances sensitivity to chemotherapeutic agents in CD133+ stem-like cancer cells

**Fig 4:**
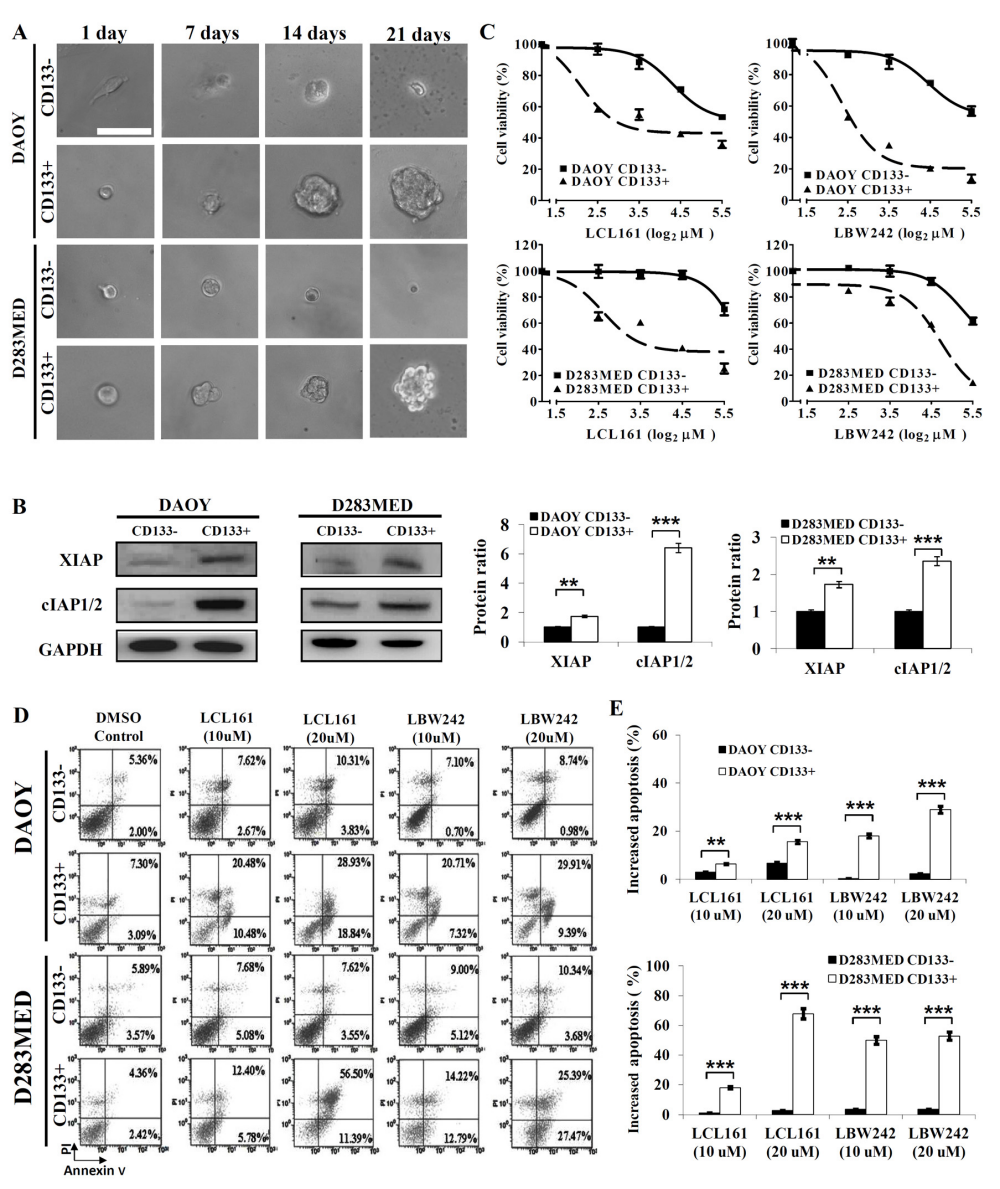
High levels of XIAP and cIAP1/2 in CD133+ stem-like MB cells result in high sensitivity to IAP inhibitors. (A) Both CD133- and CD133+ cells isolated from DAOY and D283MED cells were cultured in DMEM-F12 supplemented with 10ng/ml bFGF, 10ng/ml EGF, and 1% N2. Formation of neurospheres was examined by light microscopy. Bar scale represents 100 μm. (B) On day 21, the levels of XIAP and cIAP1/2 in CD133- and CD133+ cells were detected by immunoblotting and analyzed quantitatively. (C) CD133- and CD133+ cells were respectively treated with different concentrations of LCL161 or LBW242 for 72 hr. The cell viability was analyzed by MTT assay. (D)(E) Subsequent to treatment with LCL161 or LBW242 for 72 hr, apoptotic propitiations of CD133- and CD133+ cells were analyzed by Annexin V/PI and FACS, and then shown in bar graphs. Similar results were obtained by three independent experiments. (*p < 0.05, **p < 0.001, and ***p < 0.005).

**Fig 5:**
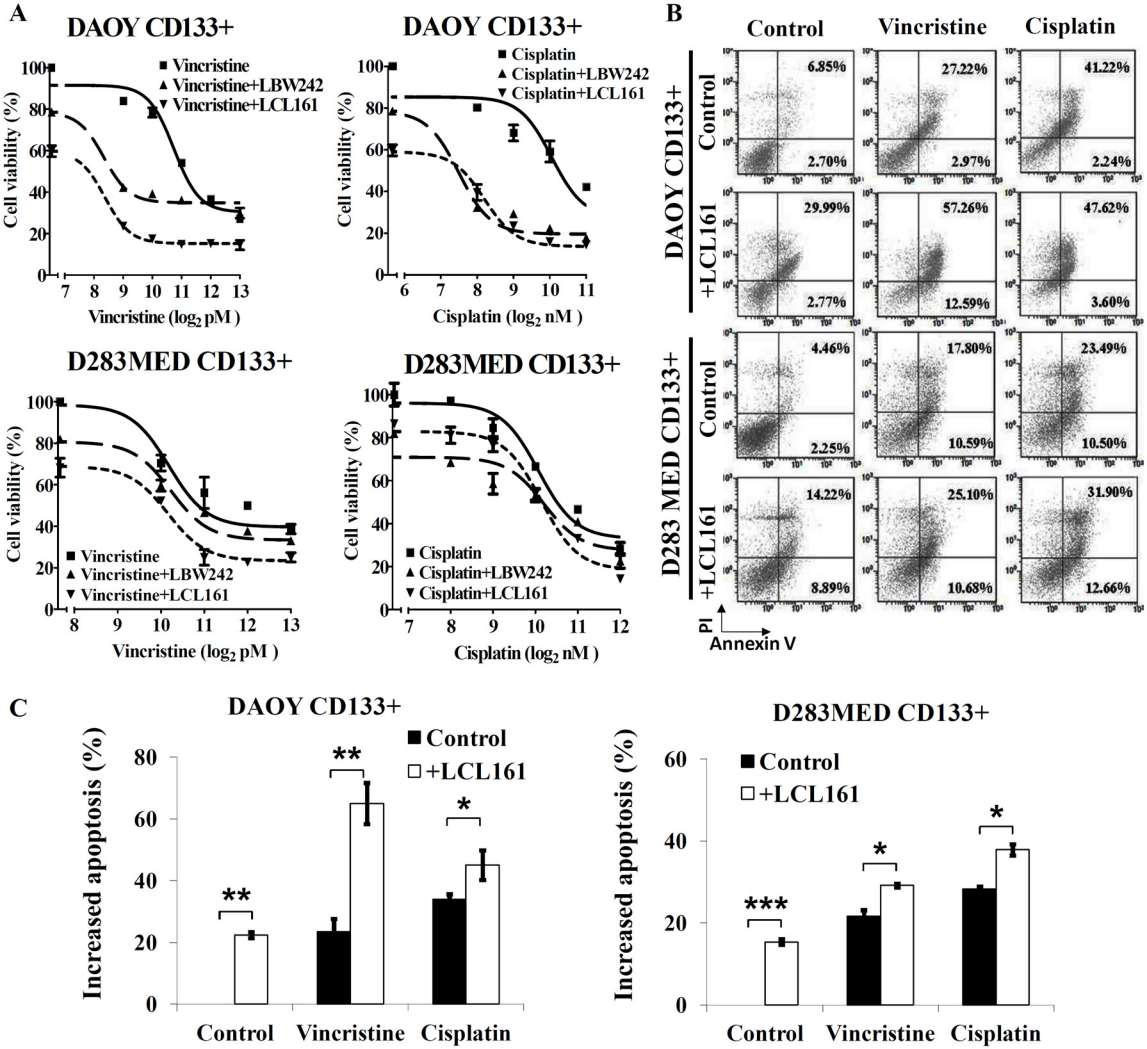
IAP inhibitors sensitize CD133+ stem-like MB cells to chemotherapeutic agents. (A) CD133+ cells isolated from DAOY and D283MED cell lines were cultured in 96-well plates and treated with different concentrations of vincristine or cisplatin combined with or without IAP inhibitors (LCL161 10 μM) for 72 hr. The cell viability was measured by MTT assay. (B)(C) Cells were treated with chemotherapeutic drug (vincristine or cisplatin) or in combination with IAP inhibitors for 72 hours. Apoptosis was detected by Annexin V/PI and FACS. The bar graphs indicate the proportion of induced apoptosis. Similar results were obtained by three independent experiments. (*p < 0.05, **p < 0.001, and ***p < 0.005).

CD133+ stem-like cells are associated with resistance to radiation and play a tumorigenic role in MB [17,24]. To identify whether MB CD133+ cells possess stemness and characteristics of CSCs, we cultured them in the presence of growth factors for three weeks. As expected, the CD133+ cells were capable of forming free-floating spherical aggregates (neurospheres) from single cells (Fig 4A). CD133+ cells expressed higher levels of stem cell markers including Nestin, SOX-2, and Oct4 than CD133- cells (S3 Fig). Interestingly, these CD133+ stem-like cells also expressed higher levels of XIAP and cIAP1/2 (Fig 4B), which corresponded to lower ED50 values for LCL161 or LBW242 compared to their CD133- counterparts (Fig 4C). Treatment with IAP inhibitors increased apoptotic (Annexin V positive) proportions in a dose-dependent manner and induced a 3 to 10-fold increase in CD133+ stem-like cells relative to MB CD133- cells (Fig 4D and 4E). Vincristine- or cisplatin-combined with IAP inhibitors tremendously attenuated proliferative activity and enhanced apoptosis of CD133+ DAOY cells and CD133+ D283MED cells (Fig 5A–5C). Combination with IAP inhibitors led to drastic decreases in ED50 values of cisplatin and vincristine in CD133+ DAOY cells (S1 Table). These results suggest that IAP inhibitors diminish resistance to chemotherapeutic agents in both MB cells and CD133+ MB cells.

**Fig 6:**
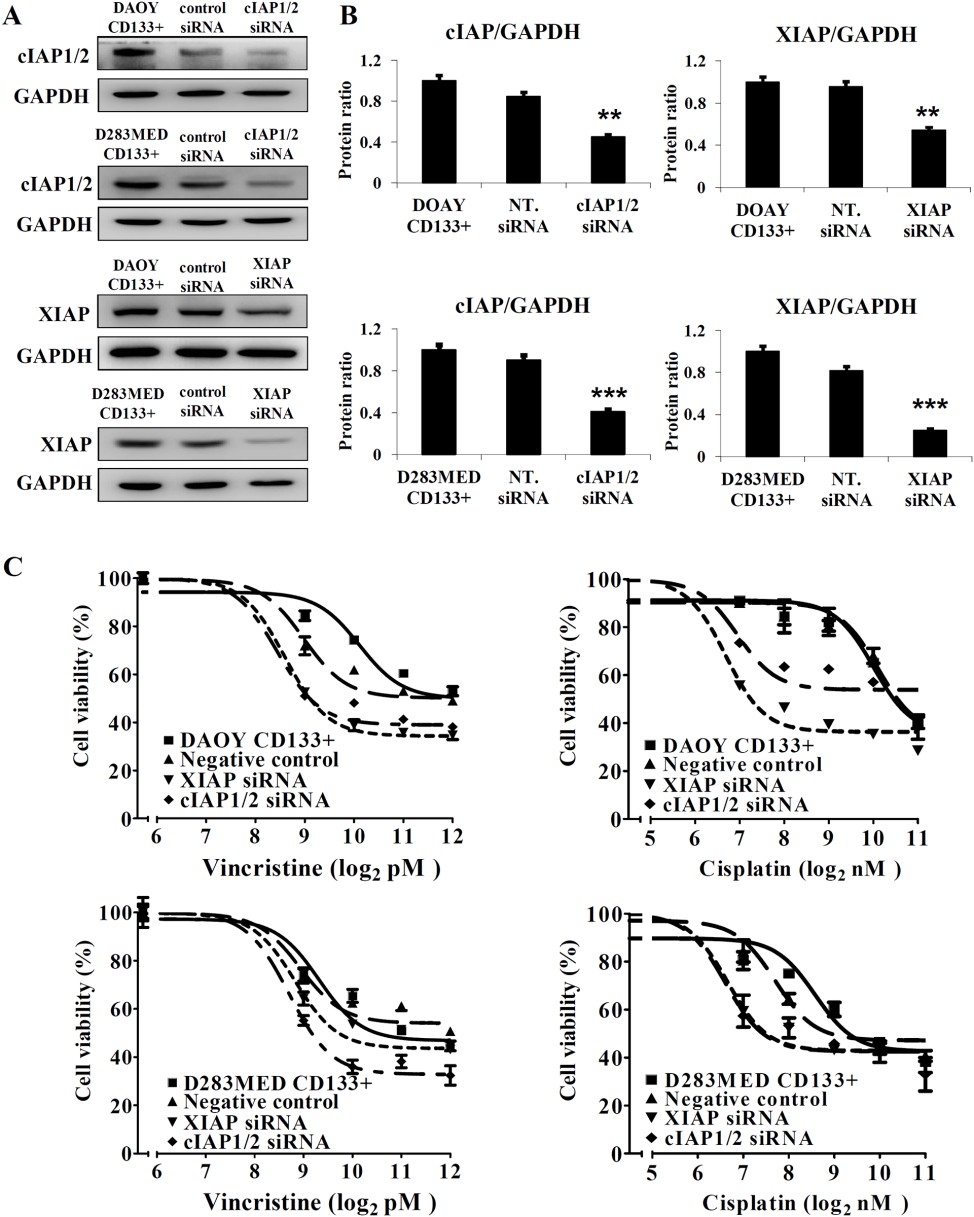
Knockdown of cIAP or XIAP enhances inhibitory effect of chemotherapies on CD133+ cell viability as well as IAP inhibitors. CD133+ MB cells were transfected with non-targeting (NT, control) siRNA and siRNA against cIAP1/2 or XIAP. The levels of XIAP and cIAP were determined using immunoblotting analysis (A), and quantified by densitometry and then shown in the bar graphs (B). (C) The transfectants were treated with different concentrations of vincristine or cisplatin for 72 hr, and cell viability was analyzed by MTT assay. The data are presented as the mean ± SEM of triplicates (*p < 0.05, **p < 0.01, and ***p < 0.005).

As mentioned above, we demonstrated that IAP inhibitors have promising anti-tumor effects on both MB cells and CD133+ cancer stem-like cells. To determine whether silencing XIAP or cIAP1/2 enhances the sensitivity to chemotherapeutic agents in CD133+ stem-like cells as well as IAP inhibitors, CD133+ cells were transfected with siRNAs against IAPs followed by treatment with vincristine or cisplatin for 72 hr. Knockdown efficiency of siRNA was confirmed by immunoblotting (Fig 6A and 6B). The results demonstrated that knockdown of XIAP or cIAP1/2 sensitizes CD133+ stem-like cells to treatment with cisplatin or vincristine (Fig 6C).

## Discussion

The primary treatment of MB consists of maximal tumor resection followed by craniospinal radiotherapy and chemotherapy [25,26]. Several chemotherapeutic agents, including vincristine and cisplatin have been used against aggressive neoplasms [8–10,27]. However, cancer cells usually develop resistance to conventional chemodrugs and radiation, which limits the therapeutic effectiveness of these drugs. Vincristine is known to be neurotoxic and cause peripheral neuropathy [28,29]. Cisplatin has been shown to elicit nephrotoxicity and ototoxicity [30–32]. The toxicity aspects of these drugs cause detrimental side effects to patients. Therefore, it is important to develop a new strategy that can lower dosage effectively and alleviate the side-effects for cancer patients.

IAPs are a family of cell death regulators, which can block apoptosis by binding to caspases or interfering in apoptotic signaling. IAPs are essential to ensure cell survival and prevent uncontrolled activation of the apoptotic protease caspases. Previous studies pointed out that orally bioavailable second mitochondria-derived activator of caspases (SMAC) mimetics, LCL161 and LBW242, can antagonize IAPs, induce dysfunction of IAPs, and subsequently increase caspase activity [22,33–35]. The combination of LBW242 and temozolomide exhibited a synergistic suppression of tumor growth in glioblastoma models [12,22]. Synergistic effects of IAP inhibitor LCL161 and paclitaxel were also seen in hepatocellular carcinoma cells [36]. Keating *et al* also used LBW242 in combination with cisplatin as well as radiation and showed a benefit from the combined therapy in MB cell lines [23]. In our present study, we firstly uncovered that XIAP expression appears in 75% MB tissues but cannot be observed in normal brain tissues (Fig 1A–1D). XIAP and cIAP1/2 expressions were higher in both DAOY and D283MED cell lines compared to normal human astrocyte cells (HA-h) (Fig 1E). Moreover, we found XIAP and cIAP1/2 expression to be robust in MB cells subsequent to treatment with cisplatin and vincristine (Fig 1G). We show that vincristine or cisplatin in combination with an IAP inhibitor had a synergistic effect on MB cells.

Most of the effective cytotoxic agents damage tumor cells by triggering programmed cell death pathways [7,37]. Apoptosis (type I programmed cell death), the most common and well-known programmed cell death, is triggered in response to several anti-cancer agents [38]. On the other hand, autophagic cell death is defined as type II programmed cell death. Autophagy is initiated when cells suffer environmental stressors such as radiation stimulation, virus infection, nutrient starvation, and cytotoxic agents [39–42]. This autophagic process is helpful for cancer cells to maintain cellular energy and cope with diverse stress conditions [43,44]. The role of autophagy in cancer is still a topic of intense debate. Although autophagy is initiated as a protective response to stress, the constitutive activation of autophagy can lead to irreversible cell death by excessive degradation of essential cellular components [45]. In this study, we demonstrated that combination of an IAP inhibitor and chemotherapeutic agents can induce apoptosis through activation of caspase-3 and autophagy simultaneously (Figs 2 and 3). Furthermore, we used the autophagy inhibitor CQ to attenuate combination-induced apoptosis and verify the therapeutic mechanism (Fig 3). Therefore, we believe that these treatments trigger autophagy-dependent cell death. Currently, a few articles reported the relationship between IAPs and autophagy in cancer [46–49], and one study conducted by Huang *et al* showed that XIAP-suppressed autophagy through the XIAP-Mdm2-p53 pathway likely plays a significant role in promoting human tumor formation [46]. Kumar *et al* reported that Rottlerin induced autophagosome formation through conversion of LC3-I to LC3-II, up-regulation of Atg12 and Beclin-1, and inhibition of anti-apoptotic proteins including Bcl-2, Bcl-xL, XIAP, and cIAP-1 in breast cancer stem cells [47]. Our data showed that IAP inhibitors can induce autophagy and are associated with conversion of LC3-I to LC3-II in MB cells. These findings indicate that IAP inhibitors utilize multiple mechanisms of action, specifically apoptosis and autophagy to eliminate MB cells.

The sub-group of cancer cells called tumor-initiating or cancer stem cells (CSCs) involved in tumorigenesis, progression, chemoradioresistance, and recurrence of cancer, can significantly interfere with cancer therapy. Therefore, therapies targeting CSCs are likely to become an effective anticancer strategy. Singh *et al* reported that CD133+⁄ Nestin+ MB cells have the capacity for cell proliferation, self-renewal, and differentiation *in vitro* [50] and CD133+ MB cells were shown to result in tumor initiation *in vivo* using xenograft models [24]. Based on this evidence, CD133 is regarded as cancer stem cell marker in brain tumors. More recent studies have used CD133 to isolate stem/stem-like cells from MB cell lines and tumors [51–53]. We established CD133+ stem-like cells from DAOY and D283MED cell lines, and found that these CD133+ cancer stem-like MB cells express robust levels of XIAP and cIAP1/2 (Fig 4). In addition, LCL161 or LBW242 triggered cell death in CD133+ stem-like MB cells more effectively than in CD133- MB cells. To our knowledge, this is the first study to show the potential efficacy of targeting the IAPs as a therapeutic strategy in CD133+ cancer stem-like cells. As mentioned, these CD133+ MB cells have been reported to be more resistant to radiation [21]. Hence, chemotherapy combined with IAP inhibitors may provide a valid strategy to diminish CD133+ stem-like cells and prevent treatment resistance.

In summary, we demonstrate the synergistic effect of vincristine or cisplatin on MB cells when combined with IAP inhibitors (LCL161 or LBW242). Both IAP inhibitors, LCL161 and LBW242, are able to enhance cell death in MB cells through concomitant induction of caspase-dependent apoptosis and autophagy, which can contribute to a significant increase in cancer cell death. These results provide new information on the effects of IAP inhibitors on MB and shed light on the effectiveness of these drugs for the treatment of MB cells and cancer stem-like MB cells.

## Supporting Information

S1 Fig Combination of IAP inhibitors and chemotherapies has a mild effect on normal astrocytes. HA-h cells (normal astrocytes) were treated with different concentrations of vincristine or cisplatin combined with LCL161 (10 μM) or LBW242 (10 μM) or DMSO. The cell viability was measured by MTT assay after treatments for 72 hr. The data are represented as the mean ± SEM of triplicates.
(TIF)

S2 Fig Caspase-3 inhibition reverses combination treatment-induced cell apoptosis in MB cells. (A) DAOY and D283MED cells were treated with combination therapy (vincristine or cisplatin plus 10 μM LCL161) or DMSO /and or caspase-3 inhibitor (40 μM Z-DEVD-FMK) for 72 hr. Cell apoptosis was detected by Annexin V/PI and FACS. (B) The bar graphs illustrate the proportion of induced apoptosis (Annexin V positive cells). Similar results were obtained in 3-independent experiments. (*p<0.05, **p<0.001, and ***p<0.005).
(TIF)

S3 Fig Expressions of cancer stem cell markers appear in CD133+ MB cells. CD133+ MB cells expressed higher cancer stem cell markers including Nestin, CD44, SOX2, and Oct4 relative to their CD133- cells and parental cells. (*p < 0.05, **p < 0.001, and ***p < 0.005).
(TIF)

S1 Table ED50 values of chemotherapeutic agents or in combination of IAP inhibitors in CD133+ cells. (DOC)
