# Supplementary figures and images for: Blockade of Inhibitors of Apoptosis Proteins in Combination with Conventional Chemotherapy Leads to Synergistic Antitumor Activity in Medulloblastoma and Cancer Stem-Like Cells
Source: PLoS One. 2016 Aug 18;11(8):e0161299. doi: 10.1371/journal.pone.0161299 (PMC4990200; doi:10.1371/journal.pone.0161299)

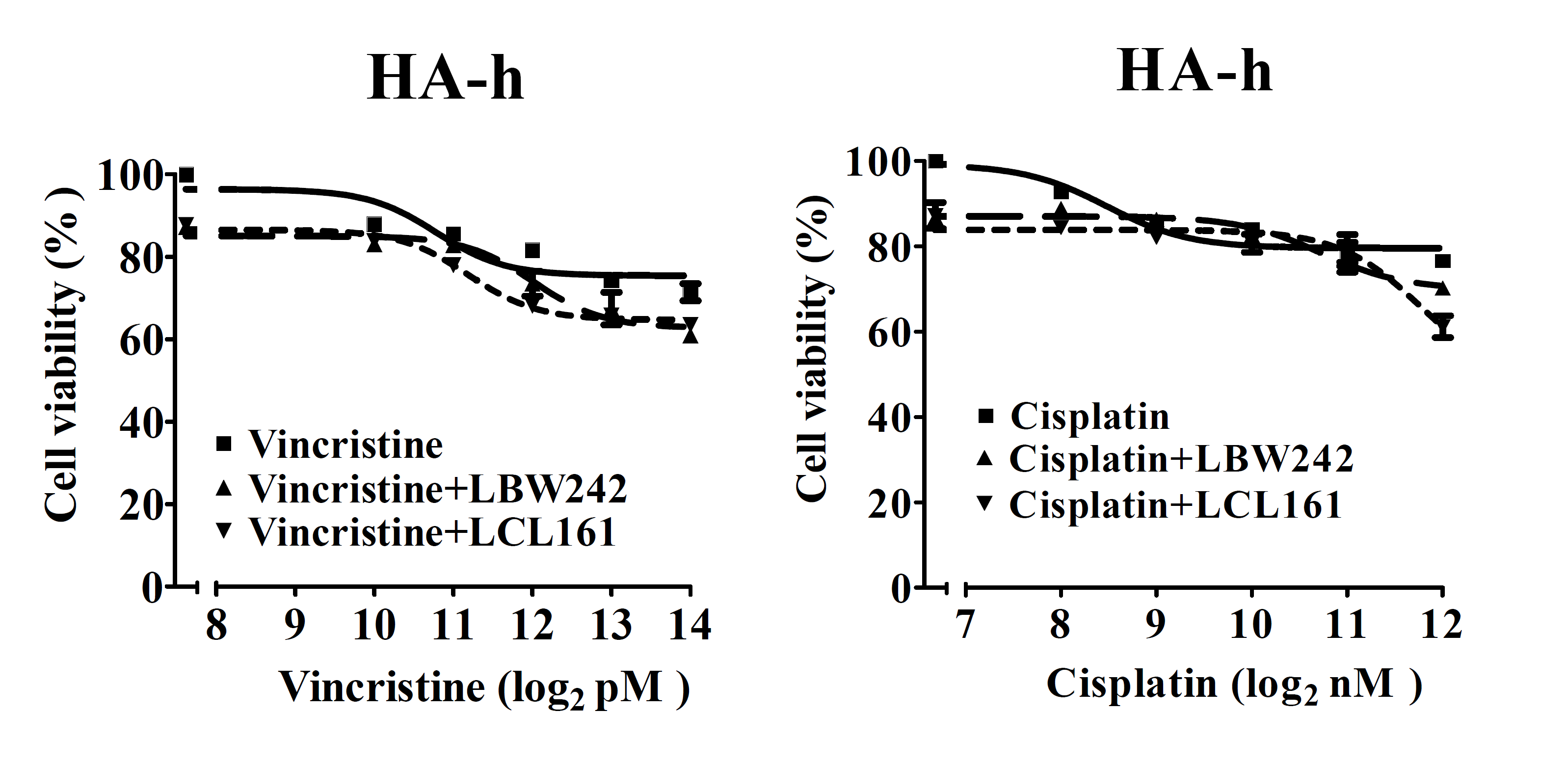

Supplement: S1 Fig — HA-h cells (normal astrocytes) were treated with different concentrations of vincristine or cisplatin combined with LCL161 (10 μM) or LBW242 (10 μM) or DMSO. The cell viability was measured by MTT assay after treatments for 72 hr. The data are represented as the mean ± SEM of triplicates. (TIF) [file pone.0161299.s001.tif]

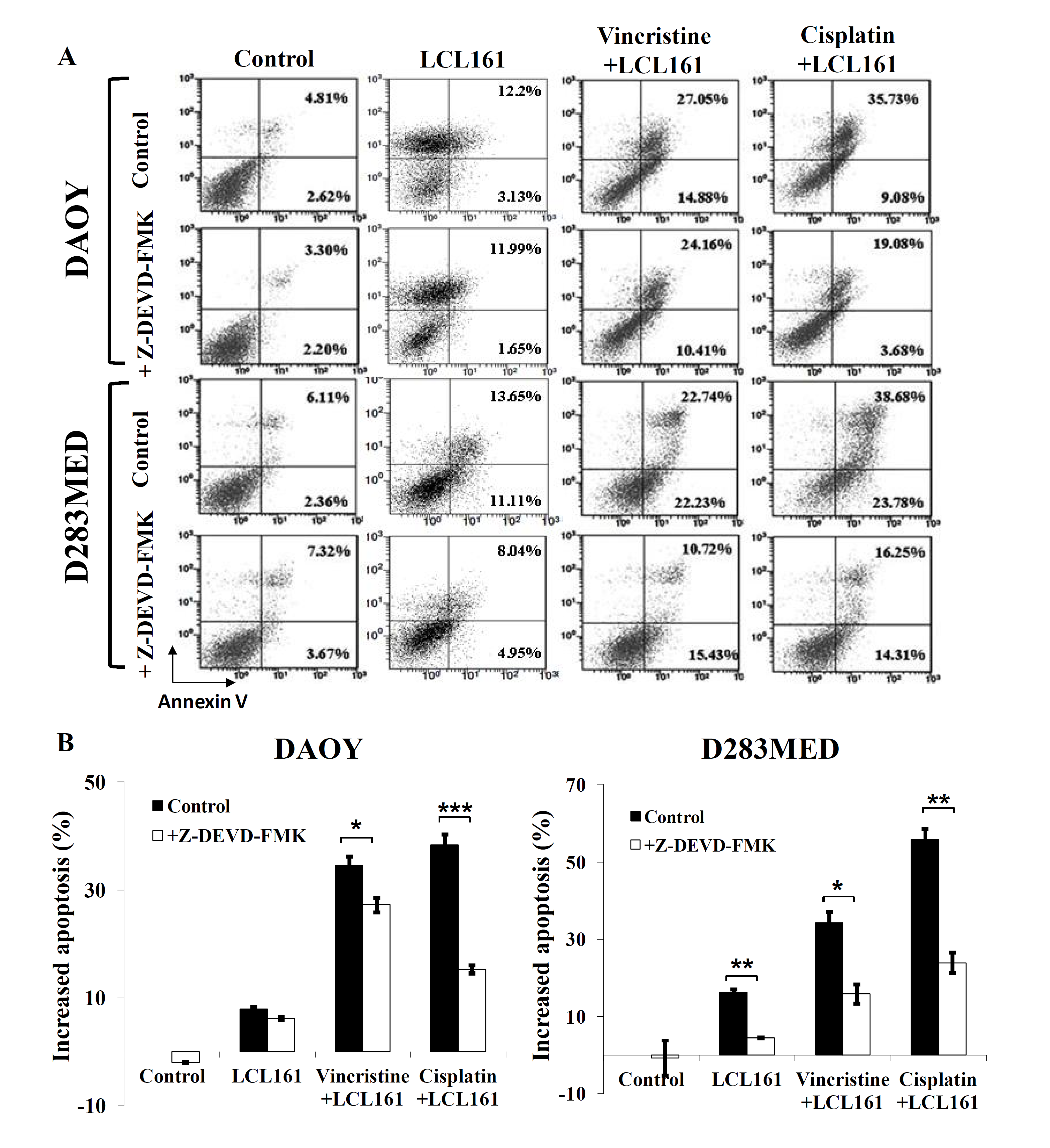

Supplement: S2 Fig — (A) DAOY and D283MED cells were treated with combination therapy (vincristine or cisplatin plus 10 μM LCL161) or DMSO /and or caspase-3 inhibitor (40 μM Z-DEVD-FMK) for 72 hr. Cell apoptosis was detected by Annexin V/PI and FACS. (B) The bar graphs illustrate the proportion of induced apoptosis (Annexin V positive cells). Similar results were obtained in 3-independent experiments. (*p<0.05, **p<0.001, and ***p<0.005). (TIF) [file pone.0161299.s002.tif]

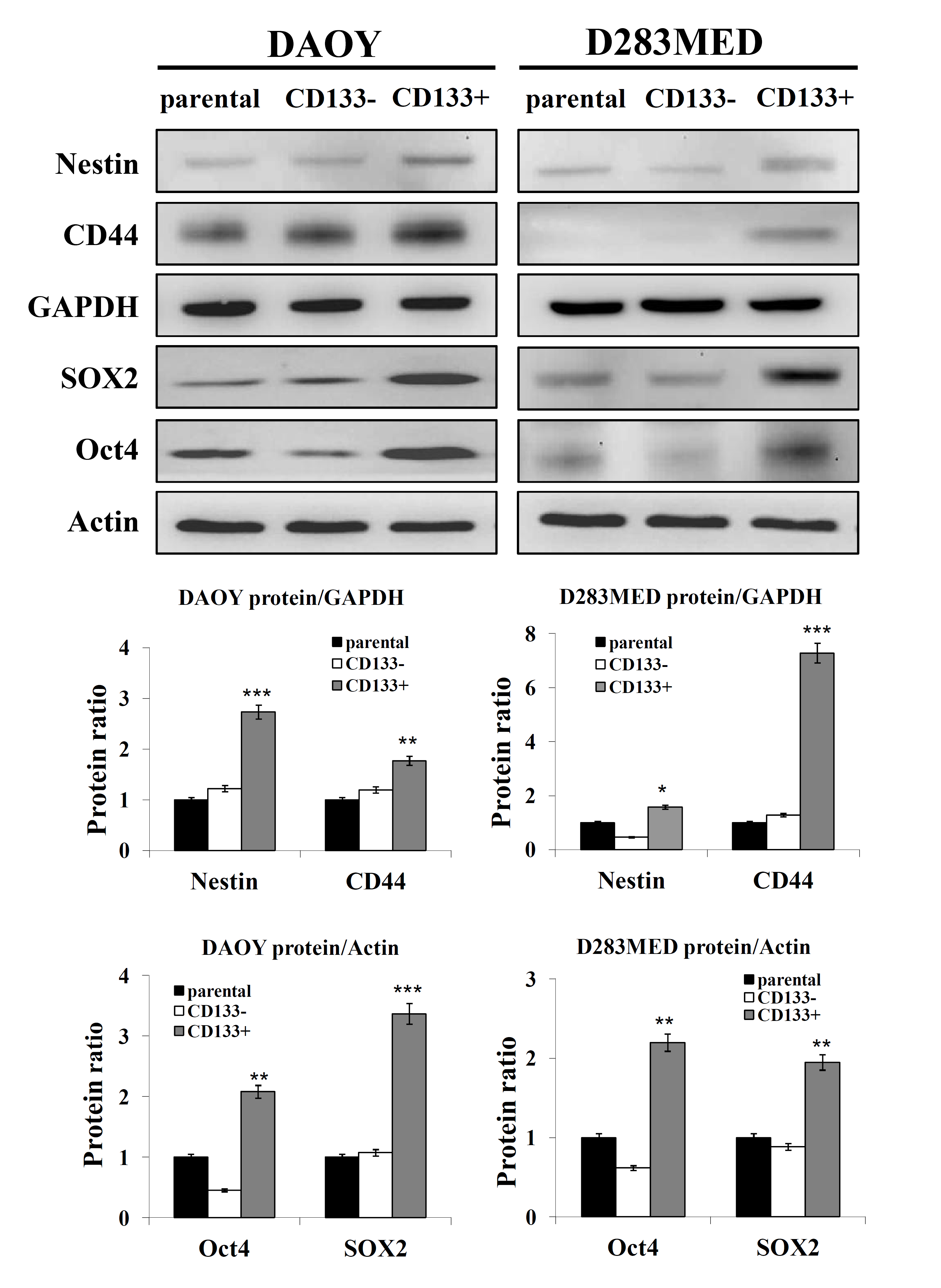

Supplement: S3 Fig — CD133+ MB cells expressed higher cancer stem cell markers including Nestin, CD44, SOX2, and Oct4 relative to their CD133- cells and parental cells. (*p < 0.05, **p < 0.001, and ***p < 0.005). (TIF) [file pone.0161299.s003.tif]
