# Supplementary material for: Blockade of Inhibitors of Apoptosis Proteins in Combination with Conventional Chemotherapy Leads to Synergistic Antitumor Activity in Medulloblastoma and Cancer Stem-Like Cells
Source: PLoS One. 2016 Aug 18;11(8):e0161299. doi: 10.1371/journal.pone.0161299 (PMC4990200; doi:10.1371/journal.pone.0161299)
Supplement: S1 Table — (DOC) [file pone.0161299.s004.doc]

| **S1 Table. ED50 of vincristine or cisplatin with or without IAP inhibitors on DAOY CD133+ and D283MED CD133+ cells.** | | | |
| --- | --- | --- | --- |
| **Cell line** | **Treatment** | **ED50** | **p-value*** |
| DAOY CD133+ | Vincristine | 6.10±0.49nM |  |
|  | Vincristine+LBW242 | 0.91±0.06nM | 0.0045 |
|  | Vincristine+LCL161 | 0.15±0.01nM | 0.0034 |
|  | Cisplatin | 1.92±0.18uM |  |
|  | Cisplatin+LBW242 | 0.19±0.07uM | 0.0062 |
|  | Cisplatin+LCL161 | 0.34±0.13uM | 0.0097 |
| D283MED CD133+ | Vincristine | 5.0±0.11nM |  |
|  | Vincristine+LBW242 | 2.16±0.03nM | 0.0008 |
|  | Vincristine+LCL161 | 1.35±0.01nM | 0.0001 |
|  | Cisplatin | 2.29±0.01uM |  |
|  | Cisplatin+LBW242 | 1.57±0.03uM | 0.0015 |
|  | Cisplatin+LCL161 | 0.2±0.02uM | 0.0001 |
| *Comparing to vincristine or ciaplatin without IAP inhibitors | | | |
